# Conspiracy Mentality in Post-Conflict Societies: Relations With the Ethos of Conflict and Readiness for Reconciliation

**DOI:** 10.5964/ejop.v15i1.1695

**Published:** 2019-02-28

**Authors:** Boban Petrović, Janko Međedović, Olivera Radović, Sanja Radetić Lovrić

**Affiliations:** a Institute of Criminological and Sociological Research, Belgrade, Serbia; b Department of Psychology, University of Pristina with seat in Kosovska Mitrovica, Kosovska Mitrovica, Serbia; c Department of Psychology, University of Banja Luka, Banja Luka, Bosnia and Hercegovina; University of Belgrade, Belgrade, Serbia

**Keywords:** conspiracy mentality, intergroup conflicts, post-conflict societies, Ethos of Conflict, Readiness for Reconciliation, lexical social attitudes

## Abstract

After almost 20 years since the end of the armed conflicts in former Yugoslavia, we are witnesses to the fact that the main causes of the conflicts have not been overcome. Reconciliation between ethnic groups that had been in conflict by means of economic and political cooperation, must have a psychological foundation. This study investigates the relations between Conspiracy Mentality, basic lexical social attitudes, and the factors important for Croatian-Serbian and Kosovo Albanian-Serbian reconciliation, i.e., the Ethos of Conflict and the Readiness for Reconciliation. We hypothesize that Conspiracy Mentality will predict the propensity for reconciliation over and above basic social attitudes, and that will mediate the relations between basic social attitudes and factors contributing (or preventing) reconciliation. With the samples of Serbs from Central Serbia (n = 307) and Northern Kosovo (n = 271), Conspiracy Mentality, Ethos of Conflict, Readiness for Reconciliation and five basic lexical social attitudes (Traditional Religiosity, Unmitigated Self-Interest, Communal Rationalism, Subjective Spirituality, and Inequality-Aversion) were measured. Results showed that Conspiracy Mentality is negatively related to the Readiness for Reconciliation and positively to the Ethos of Conflict. Additionally, Conspiracy Mentality predicts Ethos of Conflict over and above the basic social attitudes. Finally, Conspiracy Mentality mediates the relationships between Traditional Religiosity, Inequality-Aversion and Subjective Spirituality on the one hand, and Ethos of Conflict on the other. The results suggest that Conspiracy Mentality should be taken into consideration when creating policies and programmes focused on reconciliation.

As is well-known, Yugoslavia broke down on account of a series of very painful intergroup conflicts between its constitutive ethnicities. It began with the armed conflict between Serbs and Croats (1991-1995), and ended with the violent conflict between Serbs and Kosovo Albanians (1996-1999). These conflicts resulted in the establishment of independent national states, including Kosovo, the last post-Yugoslav internationally recognized the post-conflict state. All newly-formed national post-Yugoslav states are mutually recognized, except for Kosovo. Namely, Kosovo unilaterally declared its independence in 2008, and its status is still disputed by Serbia. Today, it could be said that the conflict between Serbs and Croats is formally finished, but the conflict between Serbs and Kosovo Albanians is, although formally finished, practically still on-going in practice (Bergmann, 2018). Currently, representatives of Serbia and Kosovo are engaged in a dialogue aimed to normalize their relations, under the patronage of the European Union.

However, almost 20 years since the end of the armed conflicts in former Yugoslavia, we are witnessing the fact that the main causes of the conflicts, especially the socio-psychological ones, have not been overcome. Dominant narratives and social representations of the conflicts, created at the time of their escalation, are still present in the public discourse in all former Yugoslav societies (Koska & Matan, 2017; Obradović, 2016; Pavasović Trošt, 2018). In the case of the Serbian-Croatian war, the dominant Croatian narrative about this conflict is that this conflict represented a legitimate international war waged to defend Croatian independence from Serbian aggression (Banjeglav, 2012), and to bring democracy and freedom to the Croatian people (Jansen, 2002); consequently, Croatian citizens with Serbian nationality are seen as collaborators with the aggressor (Koska & Matan, 2017). At the same time, the dominant Serbian narrative is that this war was an internal war in which Serbian people suffered (Subotić, 2013). In the case of the Kosovo Albanian-Serbian conflict, the dominant narratives are very different: while the Albanians justify the independence of Kosovo by their majority status, the Serbs claim their right to Kosovo as their historical heritage (Bieber, 2002; Obradović & Howarth, 2018), and the Albanians are seen as trying to ''biologically exterminate'' them from this area (Pavasović Trošt, 2018). But the dominant narrative among the Serbian people, common for all the Yugoslav wars, is the narrative about the Serbs (and Serbia) as historical victims, with the ever-present tendency to defend themselves from external threats (Obradović, 2016), including those coming from the international community, which is seen as negative, unjust and unfair towards Serbia and the Serbian people (Obradović & Howarth, 2018). These social representations of the conflicts have an important role in the creation of distrust between the ethnic groups and the prevention of reconciliation (Psaltis, Franc, Smeekes, Ioannou, & Žeželj, 2017). At the same time, these narratives about the conflicts could be considered as part of the broader concepts that represent the socio-psychological foundations of intractable conflicts, such as Ethos of Conflict (Bar-Tal, 2000, 2007), and Readiness for Reconciliation (Petrović, 2017).

## Psychological Roots of (Un)willingness to Reconcile

The Ethos of Conflict (Bar-Tal, 2000, 2007) is a system of societal beliefs that enables a society to adapt to a conflict situation, but it also prevents reconciliation between groups in conflict. The Ethos of Conflict is a system of eight societal beliefs: Justification of Goals, beliefs about Security, Delegitimization of Opponents, Positive Collective Self-View, Victimization, Patriotism, beliefs about Unity, and beliefs about Peace. Previous studies have shown that this concept and its operationalisation, developed from the socio-psychological analysis of the Israeli-Palestinian conflict, are applicable to the conflicts in former Yugoslavia, both conceptually and empirically. Conceptually, Bar-Tal and Čehajić-Clancy (2014) argued that from the late 1980s and the early 1990s, with the beginning of the Milošević and Tuđman eras in Serbia and Croatia, the collective identities of Serbs and Croats, but also of other ethnicities, changed, resulting in the rise of nationalism. These authors argued that Serbian national identity was organized on the basis of the collective victimhood issue, which implied the new societal goals in terms of territorial aspirations and relations with other Yugoslav ethnic groups. Consequently, collective victimhood and justification of new societal goals were related to the delegitimization of the other ethnic groups (Croats, Bosniaks, and Albanians), which resulted in the mobilization of both the Serbian and other the national forces, and the eruption of violent conflicts which lasted for almost a decade. Empirically, several studies have shown that the Ethos of Conflict measure can be adapted for use in researching conflicts in ex-Yugoslavia, regardless of the nationalities involved, and can be useful for understanding different social phenomena in post-Yugoslav societies like the evaluation of political parties (Međedović & Petrović, 2013), militant extremist beliefs (Međedović & Petrović, 2016), moral foundations (Međedović, Petrović, Radović, & Radetić-Lovrić, 2017), intergroup contacts (Petrović, Međedović, Radović, & Radetić-Lovrić, 2017), and the physical closeness of a conflicted context (Međedović & Petrović, 2012).

Researchers have invested efforts in understanding the psychological foundations of intergroup conflict escalation, and made significant efforts to develop psychological models of peaceful conflict resolution and intergroup reconciliation, i.e., to develop some kind of an ethos of peace (Bar-Tal, 2008). Taking into consideration different models of the psychological foundations of conflict escalation and conflict resolution, and combining the indicators of human potential for reconciliation defined in these theoretical models with empirically-derived indicators that had been taken from interviews with different people from three post-war Balkans countries - Serbia, Croatia and Bosnia and Herzegovina -, Petrović (2005, 2017) developed a model of human potential for reconciliation: Readiness for Reconciliation. The model proposed four factors of Readiness for Reconciliation: Trust, Cooperation, Forgiveness, and Rehumanization. However, these four factors are highly interrelated, and Readiness for Reconciliation could be interpreted as a general disposition to accept ideas and actions that lead to reconciliation and the ability to resist those leading to the conflict prolongation (Petrović, 2017). Some recent studies, independently of this model, have shown that processes described in Readiness for Reconciliation, such as trustworthiness, cooperativeness, etc., are of great importance for building social trust in the post-war period (Kijewski & Freitag, 2018).

These two concepts and, consequently, their operationalizations, clearly referred to the human potentials for reconciliation after conflict on the one hand, and for conflict prolongation and re-escalation on the other. However, previous information about their relations (Petrović, 2005) refers to the negative correlation of Readiness for Reconciliation with some of the proxy measures of Ethos of Conflict beliefs, like blind patriotism, a positive image of the in-group and negative stereotypes about outgroups (Delegitimization represents an extreme case of stereotyping, see Bar-Tal, 1989). Therefore, it is reasonable to expect high negative correlations between Ethos of Conflict and Readiness for Reconciliation. However, it could mean that these two constructs are redundant, and it might be questioned why both of these measures are included in the study. The reasons for this are multiple, but the principle one is that they include and, consequently, measure different processes of relevance to understanding the psychological capacities for reconciliation: while Ethos of Conflict is focused on the perception of one's in-group and out-groups (i.e., Positive Collective Self-View, Delegitimization of Opponents, beliefs about Unity, etc.), Readiness for Reconciliation is more focused on relationships between groups (i.e., cooperation, forgiveness, etc.). Consequently, measuring both constructs will encompass a wider range of variables important for understanding the psychological foundations for reconciliation in the post-conflict period.

The ethos of Conflict and Readiness for Reconciliation also share their strong relations with social attitudes. More concretely, Ethos of Conflict was positively associated with authoritarianism, conservatism, religiosity, traditionalism, and nationalism, and negatively with liberal and humanitarian social attitudes (e.g., Bar-Tal et al., 2012; Međedović & Petrović, 2016; Porat, Halperin, & Bar-Tal, 2015). Readiness for Reconciliation was also strongly related to these social attitudes, but in quite the opposite way (Petrović, 2005). Most of the research of Ethos of Conflict and its associations with social attitudes in the post-Yugoslav (i.e., Serbian) context has investigated the relations of Ethos of Conflict with the basic lexical social attitudes – i.e., with the “isms” (Saucier, 2000, 2013). This comprehensive model of basic social attitudes was developed through application of the lexical hypothesis paradigm in the field of social attitudes and beliefs. More specifically, Saucier’s lexical analysis of the dictionary-terms which ended with ‘‘ism‘‘ (communism, capitalism, conservatism, liberalism, etc.) resulted in five broad factors of social attitudes and beliefs: Traditional Religiosity, Unmitigated Self-Interest (i.e., selfish materialism), Communal Rationalism (i.e., humanism and respect for democratic values), Subjective Spirituality and Inequality-Aversion (i.e., Egalitarianism). Similarly, as in the case of classic, non-lexical attitudes, the Ethos of Conflict was positively related to Traditional Religiosity, Unmitigated Self-Interest, Subjective Spirituality, and, unexpectedly, Inequality-Aversion, while it was negatively associated with humanitarian social attitudes (Međedović & Petrović, 2013; Petrović, 2016; Petrović & Međedović, 2011). As was expected, Readiness for Reconciliation made a pattern with basic lexical social attitudes opposite to what was obtained with Ethos of Conflict (Petrović et al., 2017).

## Conspiracy Mentality and (Un)willingness to Reconcile

The general propensity to endorse conspiracy theories - in other words, Conspiracy Mentality - represents a phenomenon where some people “attribute significant events to the intentional actions of ill-intentioned mean-intending groups of individuals who are sufficiently powerful to carry out the suspected conspirational act”, to whatever happens in a particular society, even war, poverty, unemployment, etc. (Imhoff & Bruder, 2014). The propensity to believe in conspiracy theories has been often stimulated during societal crises, when people experience feelings of uncertainty, fear, lack of control (van Prooijen & Douglas, 2017). In this context, Conspiracy Mentality reflects some of the fundamental human motivations: to understand and to control one’s environment, to be safe and to maintain a positive image of the self and the social group (Douglas, Sutton, & Cichocka, 2017). Consequently, Conspiracy Mentality could have an impact on relations between social groups primarily through favouring distinctions between in-groups and out-groups (Cichocka, Marchlewska, Golec de Zavala, & Olechowski, 2016; Sapountzis & Condor, 2013) and strengthening and justifying hostility toward out-groups (Pruitt, 1987; Sedek & Bilewicz, 2015). For example, Mashuri and Zaduqisti (2014) found that there were clear patterns of relations between social identification, beliefs in conspiracy theories and out-group derogation. Out-group derogation mediated the effect of social identification on belief in conspiracy theories only when in-group members perceived the out-group members as highly threatening to their identity. It is important to note that during the conflicts in former Yugoslavia in 1990s, Serbia was a fertile ground for the development of numerous conspiracy theories directed toward other parties in the conflicts, including the ''world conspiracy elite'' aiming to create a ‘’New World Order’’ and to destroy Yugoslavia and the Serbian people, since the Serbs were in conflict with practically all the other Yugoslavian ethnicities, and with NATO (Byford, 2002, 2006; Byford & Billig, 2001).

Some authors have argued that Conspiracy Mentality could be considered as a generalized political attitudinal orientation reflecting prejudices against high-powered, less likable and more threatening social groups (Imhoff & Bruder, 2014). Conspiracy Mentality is positively related to some of the basic social attitudes, primarily with authoritarianism (Bruder, Haffke, Neave, Nouripanah, & Imhoff, 2013; Grzesiak-Feldman & Irzycka, 2009; Richey, 2017). In addition, Conspiracy Mentality, like other basic social attitudes (authoritarianism, conservatism, etc.) is positively related to undemocratic political orientation, xenophobia and prejudice (Gyárfášová, Krekó, Mesežnikov, Molnár, & Morris, 2013), social identity, perception of intergroup threat and out-group derogation (Mashuri & Zaduqisti, 2014; Milošević-Đorđević & Žeželj, 2017a). Furthermore, it seems that the general propensity to endorse conspiracy theories and Ethos of Conflict have significant similarities: both can be developed when conflicts are escalating, reflecting the needs to control the environment, favoring a positive in-group self-view, derogating out-group members and justifying the hostility toward them. Finally, both could possibly be factors hindering reconciliation.

## The Present Study

Previous findings indicate that basic social attitudes play an important role in understanding the processes of reconciliation (or unwillingness to reconcile; see, for example, Bar-Tal et al., 2012; Međedović & Petrović, 2013; Petrović, 2005). In addition, Conspiracy Mentality has a nomological network similar to social attitudes (e.g., Bruder et al., 2013), and has some conceptual similarities with Ethos of Conflict. The objective of this research is to examine the role of Conspiracy Mentality in understanding the (un)willingness to reconcile over and above the basic social attitudes. More specifically, this study investigates the relations between Conspiracy Mentality, basic social attitudes, and factors important for Croatian-Serbian and Kosovo Albanian-Serbian reconciliation, i.e., Ethos of Conflict and Readiness for Reconciliation. On the basis of the results of previous studies showing positive relations between Conspiracy Mentality on one side, and authoritarianism, the need for national security, social identity salience, demonization of the other, intergroup threat, etc., on the other, we have made several hypotheses.

Following previously argued conceptual similarities between Conspiracy Mentality and Ethos of Conflict (see, for example, Bruder et al., 2013; Mashuri & Zaduqisti, 2014; Pruitt, 1987), we hypothesize that *Conspiracy Mentality will be positively related to the Ethos of Conflict and negatively to the Readiness for Reconciliation (Hypothesis 1).*

Although some authors claim that Conspiracy Mentality could be understood as a generalized attitudinal orientation (Imhoff & Bruder, 2014), previous research has shown that Conspiracy Mentality is associated with, but not reducible, to basic social attitudes like authoritarianism (the coefficient of correlation between them is about .30; see, e.g., Bruder et al., 2013; Grzesiak-Feldman & Irzycka, 2009). In addition, as has previously been shown, both proneness to believe in conspiracy theories and social attitudes are linked to psychological factors important for conflict escalation, including Ethos of Conflict. It should also be borne in mind here that Traditional Religiosity, Unmitigated Self-Interest, Subjective Spirituality, and Inequality-Aversion are positively associated with the Ethos of Conflict, while Communal Rationalism has negative relations with it (Međedović & Petrović, 2013; Petrović, 2016; Petrović & Međedović, 2011). Therefore, although it could be assumed that Conspiracy Mentality and conservative social attitudes like Traditional Religiosity share some percent of the variance, they are independent. Consequently, we could expect that *Conspiracy Mentality will predict the Ethos of Conflict and Readiness for Reconciliation over and above basic social attitudes – specifically, we expect that all of them could make a significant contribution, but that more important predictors could be Traditional Religiosity, Communal Rationalism, and Inequality-Aversion (see, for example,* *Petrović, 2016**) (Hypothesis 2).*

Basic social attitudes like authoritarianism and conservatism are the main predictors of Conspiracy Mentality (Richey, 2017), but also of the abovementioned psychological foundations for reconciliation (Petrović, 2005; Međedović & Petrović, 2016). Besides the conceptual similarities with Ethos of Conflict, there is empirical evidence that Conspiracy Mentality has a mobilising role in times of political change, through mapping social identity, justifying hostility in conflicts (Milošević-Đorđević & Žeželj, 2017b; Sedek & Bilewicz, 2015), and facilitating escalations of conflict (Pruitt, 1987). In the former Yugoslav post-conflict societies, true reconciliation has not occurred even after almost 20 years since the end of the armed conflict. It has been suggested that Ethos of Conflict and Readiness for Reconciliation are the crucial variables in this research, since they are psychological dispositions that are fundamentally important for reconciliation and an end of conflicts (see, for example, Bar-Tal et al., 2012). These are the reasons for setting up a mediation model, and to expect that *Conspiracy Mentality will mediate the relations between basic lexical social attitudes and the two forementioned factors contributing to (or preventing) reconciliation (Hypothesis 3).*

More concretely, following previous research (e.g., Petrović, 2016) we expect that *Traditional Religiosity and Communal Rationalism will have direct pathways to the Ethos of Conflict and Readiness for Reconciliation, but in opposite directions (Hypothesis 3.1).* But, bearing in mind that they represent the authoritarian or humanistic attitudinal orientations respectively, we could also expect that *Conspiracy Mentality will mediate their relations (Hypothesis 3.2).* When we consider the role of Inequality-Aversion, there are more difficulties in making a clear hypothesis – starting from the basic assumption that it represents an egalitarian orientation, we could expect *a positive direct effect on the Ethos of Conflict and a negative effect on the Readiness for Reconciliation (Hypothesis 3.3).* However, having in mind also the existing empirical material (e.g., Petrović, 2016), *the expected effects could be quite the opposite (Hypothesis 3.4)*. In the last case, the mediating effect of the Conspiracy Mentality is more likely. In the case of Unmitigated Self-Interest and Subjective Spirituality, it is difficult to make any predictions, because previous findings regarding their relations with the criterion variables are not consistent. However, conceptually we could expect the *positive paths of Unmitigated Self-Interest and Subjective Spirituality toward the Ethos of Conflict and their negative paths toward the Readiness for Reconciliation (Hypothesis 3.5).*

Finally, this research is focused on the conflicts between Serbs and Croats and Serbs and Kosovo Albanians, and collection of data provided by samples of Serbs from Central Serbia and Northern Kosovo. These two conflicts have certain similarities and differences, and, as we mentioned earlier, there are different social representations of these conflicts among Serbian people. Actually, the biggest difference is that the conflict between Serbs and Croats ended in late 1995, and, despite occasional incidents, relations between Serbia and Croatia (and Serbs and Croats) have been relatively stable since the end of the war, while the armed conflict between Serbs and Kosovo Albanians was formally finished in 1999, but has not yet been completely resolved, as sometimes becomes apparent in violent incidents between the group members. It is important to note here that some recent studies have shown that conservative social attitudes and Ethos of Conflict are more pronounced in Kosovo than in Serbia (Međedović & Petrović, 2012). In this context, *we expect Serbs from Northern Kosovo to have a more pronounced Ethos of Conflict, but less Readiness for Reconciliation with Albanians than respondents from Central Serbia in terms of their relations with Croats (Hypothesis 4.1)*. Also, *Northern Kosovo Serbs will have higher scores on Traditional Religiosity and lower on Communal Rationalism than those from Central Serbia (Hypothesis 4.2)*. Following previous findings that suggested that Conspiracy Mentality might have a facilitating role in conflict escalation (Pruitt, 1987), we could expect that *respondents from Northern Kosovo will have a higher score on Conspiracy Mentality than those from Central Serbia. However, although both conflicts taken into consideration in this research were formally finished, they are still on-going and have resulted in occasional incidents. It suggested that we could expect higher scores on Conspiracy Mentality on both subsamples, as well as that there are no differences between them (Hypothesis 4.3)*.

In terms of a proposed mediation model, we expect that *Traditional Religiosity and Communal Rationalism will have both direct and indirect effects (mediated by Conspiracy Mentality) on Ethos of Conflict and Readiness for Reconciliation (Hypothesis 5.1).* Despite the proposed differences between Serbs from Northern Kosovo and Central Serbia, the fact is that basic social attitudes (and probably Conspiracy Mentality) have power to predict Ethos of Conflict in different contexts and on different samples (Bar-Tal et al., 2012; Petrović, 2016; Petrović & Međedović, 2011). This fact leads to an hypothesis about the cross-samples stability of the proposed model, i.e., that *the proposed model will be invariant through the samples (Hypothesis 5.2)*.

## Method

### Participants

The study was conducted on a total sample of 578 respondents, consisting of two subsamples. One was from Central Serbia, *n* = 307, 63.6% females; average age 39.49 (*SD* = 15.56), and on average 13.83 years of education (*SD* = 2.64). The other subsample was from Kosovo, i.e., from Kosovska Mitrovica, *n* = 271, 61.8% females; average age 39.36 (*SD* = 14.33), and on average 12.95 years of education (*SD* = 2.49). Participants were students of the University of Belgrade and the University of Priština with its seat in Kosovska Mitrovica, and their parents (58.3% and 66.2% of parents in Serbian and Kosovo samples, respectively). All participants were Serbs. The respondents participated voluntarily and signed informed consent forms. The students participated in the study in exchange for course credits. Data were collected during the period 2016-2018. In the subsample from Central Serbia, the target conflict was that between Serbs and Croats. In the subsample of respondents from Kosovo, the conflict between Serbs and Kosovo Albanians was targeted.

### Measures

#### The Ethos of Conflict Scale

The Ethos of Conflict Scale (EOC; Bar-Tal et al., 2012) contains 48 items, 6 per each of the 8 conflict beliefs: *Justification of Goals*, e.g., “The exclusive right of Kosovo Serbs to the land stems from it being their historical homeland”, *Beliefs about Security,* e.g., “Executing military actions is the most efficient means to eliminate threats to the country’s security”, *Delegitimization of Opponents,* e.g., “Albanians in Kosovo have always been characterised by untrustworthiness”, *Positive Collective Self-View,* e.g., “Serbs have always been known for their wisdom”, *Victimization,* e.g., “During the conflict between Serbs and Albanians in Kosovo, Serbs were usually the victims of the Albanian aggression”, *Patriotism*, e.g., “Fostering a feeling of loyalty to one’s homeland should be one of the most important goals of the educational system”, *Beliefs about Unity*, e.g., “If Serbian people are not united, they are in danger of annihilation”, and *Beliefs about Peace,* e.g., “Most Serbs have always aspired to resolve the conflict with the Albanians in Kosovo peacefully”.

#### The Readiness for Reconciliation Scale

The Readiness for Reconciliation Scale (RR; Petrović, 2005, 2017) consists of 20 items, 5 per each facet of Readiness for Reconciliation. The items that represented the four dimensions of Readiness for Reconciliation are: *Trust*, e.g., “We always turn out to have been naive and used because of being open to Them (reversed)”, *Cooperation,* e.g., “I think that cooperation with Them is necessary and of mutual benefit”, *Forgiveness*, e.g., “I am personally ready to forgive everyone”, and *Rehumanization*, e.g., “The acts of those who committed crimes do not make all of Them mean people”.

Both the Ethos of Conflict and Readiness for Reconciliation scales were adapted with respect to the conflict targeted within the subsamples. All analyses were conducted separately on these two samples.

Following previous studies (e.g., Bar-Tal et al., 2012; Petrović, 2017; Petrović & Međedović, 2011), and with the aim of testing an assumption about the unidimensionality of both Ethos of Conflict and Readiness for Reconciliation measures, series of principal component analyses were conducted, separately in each of the subsamples. The maximum likelihood method of factor extraction was used, and the principal components were rotated in the Promax position. In the cases of Trust, Cooperation, Forgiveness, and Rehumanization, the parallel analysis showed that one principal component can be extracted in both subsamples (see Table A1 in the Appendix), explaining 64.1 and 52.7 percents of the variance in Serbian and Kosovo samples, respectively (λ_Serbia_ = 2.56, λ_Kosovo_ = 2.11). In both cases, the extracted principal component was interpreted as Readiness for Reconciliation. Similarly, the principal component analysis of eight Ethos of Conflict beliefs (Table A2 in the Appendix) resulted in one principal component in both subsamples (λ_Serbia_ = 3.51, 43.9% of the variance; λ_Kosovo_ = 2.65, 33.1% of variance), which can be interpreted as the General Ethos of Conflict. Following these findings, we calculated mean scores from both of these variables and included them as criterion measures in further analysis.

#### The Survey of Dictionary-Based Isms

The Survey of Dictionary-Based Isms (SDI-25; Saucier, 2013) was used for the assessment of basic lexical social attitudes. Five basic social attitudes were measured: *Traditional Religiosity*, e.g., “I adhere to an organized religion”, *Unmitigated Self-Interest*, e.g., “What is good can only be judged by the gratification of the senses”, *Communal Rationalism,* e.g., “I am in favour of a constitutional form of government”, *Subjective Spirituality*, e.g., “There is an ideal spiritual reality that goes beyond sense experience and science and is knowable through intuition”, and *Inequality-Aversion*, e.g., “I support the rights and power of the people in their struggle against the privileged elite”.

#### The Conspiracy Mentality Questionnaire

The Conspiracy Mentality Questionnaire (CMQ; Bruder et al., 2013), a 5-item scale measuring the general Conspiracy Mentality, e.g., “I think that there are secret organizations that greatly inﬂuence political decisions”. A previous study (Milošević-Đorđević & Žeželj, 2017b) showed that the CMQ had adequate convergent validity.

All questionnaires had a joint 5-point Likert-type scale, from *1 – strongly disagree* to *5 – strongly agree*. All of the scales used in the research were back-translated into the Serbian language in cooperation with the original authors. The measures were also previously administered in Serbia, and their validity demonstrated (Međedović & Petrović, 2013; Milošević-Đorđević & Žeželj, 2017b; Petrović, 2005; Petrović & Međedović, 2017)^i^.

### Data Analysis

We have calculated the descriptive statistics and differences between groups of respondents from Central Serbia and Northern Kosovo. To determine the relations between variables, bivariate correlations were calculated and linear regression analyses were conducted. The Readiness for Reconciliation and the general Ethos of Conflict were entered as criteria, and the five basic social attitudes and Conspiracy Mentality were entered as predictor variables in regression models. The effects of gender, age, education and family status (since samples consisted of students and their parents, this variable was transformed into dummy variables prior to running regression analyses) of the respondents were controlled. To test the mediation role of Conspiracy Mentality on relations between basic social attitudes and Readiness for Reconciliation and the Ethos of Conflict across samples, multi-group path analysis was used. Several fit indices were used to determine the model fit: χ^2^, comparative fit index – CFI and root mean square error of approximation – RMSEA. Values of fit indices indicating good fit are: RMSEA < .06, and CFI > .95), and acceptable fit: RMSEA < .08, and CFI > .90 (see Hu & Bentler, 1999; Lazarević, 2008).

## Results

### Descriptive Statistics and Correlations Between Examined Variables

The descriptive statistics and differences between groups of respondents from Central Serbia and Northern Kosovo for all examined variables are shown in Table 1. As can be seen, the differences between groups are registered for almost all variables, except Inequality-Aversion. Respondents from Central Serbia scored highly on the Readiness for Reconciliation with Croats, Communal Rationalism, and Subjective Spirituality. Respondents from Kosovo had higher scores than those from Central Serbia on Ethos of Conflict toward Albanians, Traditional Religiosity, and Unmitigated Self-Interest, and lower on Conspiracy Mentality.

**Table 1:** Descriptive Statistics and Group Differences for the Examined Variables

Variable	Serbia (*n* = 307)		Kosovo (*n* = 271)		*t*(578)	*d*
	*M*	*SD*	*M*	*SD*		
RR	3.84	0.65	3.23	0.63	-11.89**	0.95
EOC	2.93	0.37	3.39	0.34	15.93**	-1.29
TRA	3.04	0.66	3.60	0.48	11.48**	-0.97
USI	2.37	0.85	2.90	0.82	7.68**	-0.63
CR	4.07	0.60	3.92	0.59	-3.34**	0.25
SS	3.23	0.75	3.02	0.61	-3.72**	0.31
IA	3.52	0.52	3.54	0.51	0.35	-0.04
CM	4.07	0.63	3.93	0.69	-2.13*	0.21

*Notes.* RR = Readiness for Reconciliation; EOC = Ethos of Conflict; TRA = Traditional Religiosity; USI = Unmitigated Self-Interest; CR = Communal Rationalism; SS = Subjective Spirituality; IA = Inequality-Aversion; CM = Conspiracy Mentality.

*p < .05. **p < .01.

The bivariate correlations between examined variables separately for two subsamples are displayed in Table 2. In both subsamples, Readiness for Reconciliation and general Ethos of Conflict were highly negatively correlated. Traditional Religiosity was also consistently correlated negatively to Readiness for Reconciliation and positively to Ethos of Conflict. Communal Rationalism was positively related to Readiness for Reconciliation, but there was no significant relation with Ethos of Conflict. It was interesting that Inequality-Aversion was positively related to Ethos of Conflict and unrelated to Readiness for Reconciliation. As expected, Conspiracy Mentality was positively correlated to Ethos of Conflict, but also with Traditional Religiosity. These findings are consistent through two subsamples.

**Table 2:** Bivariate Correlations and Reliabilities of the Examined Variables

Variable	1	2	3	4	5	6	7	8
1. RR	*.91/.78*	-.50**	-.29**	-.04	.17**	.01	-.17**	-.09
2. EOC	-.72**	*.85/.72*	.39**	.04	.09	.04	.27**	.31**
3. TRA	-.31**	.55**	*.72/.65*	.22**	.14*	.16	.14*	.23**
4. USI	.00	.01	.10	*.80/.78*	.10	.17**	.07	.04
5. CR	.13^†^	-.01	-.01	-.00	*.70/.65*	.12*	.26**	.11
6. SS	.10	-.03	-.04	.08	.05	*.67/.61*	-.04	.09
7. IA	-.12	.14^†^	.15*	-.02	.15*	.02	*.64/.61*	.24**
8. CM	-.18**	.26**	.21**	.01	-.02	.15*	.11	*.77/.71*

*Note.* RR = Readiness for Reconciliation; EOC = Ethos of Conflict; TRA = Traditional Religiosity; USI = Unmitigated Self-Interest; CR = Communal Rationalism; SS = Subjective Spirituality; IA = Inequality-Aversion; CM = Conspiracy Mentality. Correlation coefficients between examined variables from the Serbian samples (*n* = 307) are presented below, from the Kosovo samples (*n* = 271) above the diagonal. Alpha coefficients of internal consistency are shown in diagonal and separated by a slash for Serbia/Kosovo.

^†^*p* < .10. *p < .05. **p < .01.

There are some important differences between subsamples: Conspiracy Mentality was negatively related to Readiness for Reconciliation and positively to Subjective Spirituality in the Serbian sample, but in the Kosovo sample, the correlation between these two measures was not significant. But, Conspiracy Mentality correlated positively with Inequality-Aversion only in the sample from Northern Kosovo.

Coefficients of reliability (Table 2) are satisfactory or good for the majority of measures; however, in the case of Subjective Spirituality and Inequality-Aversion, in both subsamples the reliability is questionable but still acceptable. Reliability coefficients are generally slightly weaker in the Kosovo subsample than in the subsample from Central Serbia.

### Conspiracy Mentality as a Predictor of Ethos of Conflict and Readiness for Reconciliation

Results of regression analyses conducted separately on the subsamples (Table 3) showed that basic social attitudes and Conspiracy Mentality explained 14% i.e., 18% of the variance of Readiness for Reconciliation in the Serbian and Kosovo subsamples respectively, and 33% of the variance of Ethos of Conflict in the Serbian and 26% in the Kosovo subsample. In both subsamples, the strongest predictors of the Readiness for Reconciliation were low Traditional Religiosity and higher Communal Rationalism in both subsamples. Conspiracy Mentality had a significant effect on the prediction of Readiness for reconciliation only in the Serbian subsample, but, in contrast, in the Kosovo subsample, Inequality-Aversion showed significant predictive power. However, only Traditional Religiosity and Conspiracy Mentality showed significant effects in the prediction of Ethos of Conflict, consistently through the subsamples.

**Table 3:** Social Attitudes and Conspiracy Mentality as Predictors of Ethos of Conflict and Readiness for Reconciliation – Results of Regression Analyses Conducted Separately on the Samples From Serbia and Kosovo

Predictor	Serbia (*n* = 307)				Kosovo (*n* = 271)			
	Reconciliation		Ethos of Conflict		Reconciliation		Ethos of Conflict	
	β	*t*	β	*t*	β	*t*	β	*t*
Sex	-.02	-0.48	-.01	0.02	.00	0.06	-.01	-0.23
Age	.02	2.16	.14	-1.12	.05	0.35	.16	1.13
Education	.03	-0.15	-.06	-0.54	.00	0.00	.07	1.17
Family-parents	.02	-2.04	-.08	1.69	.03	0.22	-.07	-0.53
TRA	-.28	-3.75**	.51	8.41**	-.32	-5.21**	.35	6.01**
USI	.02	0.81	-.05	-1.47	.00	-0.01	-.07	-1.25
CR	.12	2.62*	-.02	-0.53	.26	4.23**	-.06	-0.96
SS	.11	2.04	-.02	-0.31	.04	0.68	.02	0.31
AI	-.09	-1.50	.04	0.12	-.20	-3.26**	.19	3.18**
CM	*-.12*	-2.05*	.15	3.55**	.00	0.06	.20	3.44**
*R*^2^	.14**		.33**		.18**		.26**	

*Note.* TRA = Traditional Religiosity; USI = Unmitigated Self-Interest; CR = Communal Rationalism; SS = Subjective Spirituality; IA = Inequality-Aversion; CM = Conspiracy Mentality.

*p < .05. **p < .01.

### Conspiracy Mentality as a Mediator Between Social Attitudes and Ethos of Conflict and Readiness for Reconciliation – Multi-Group Path Analyses

**Figure 1:**
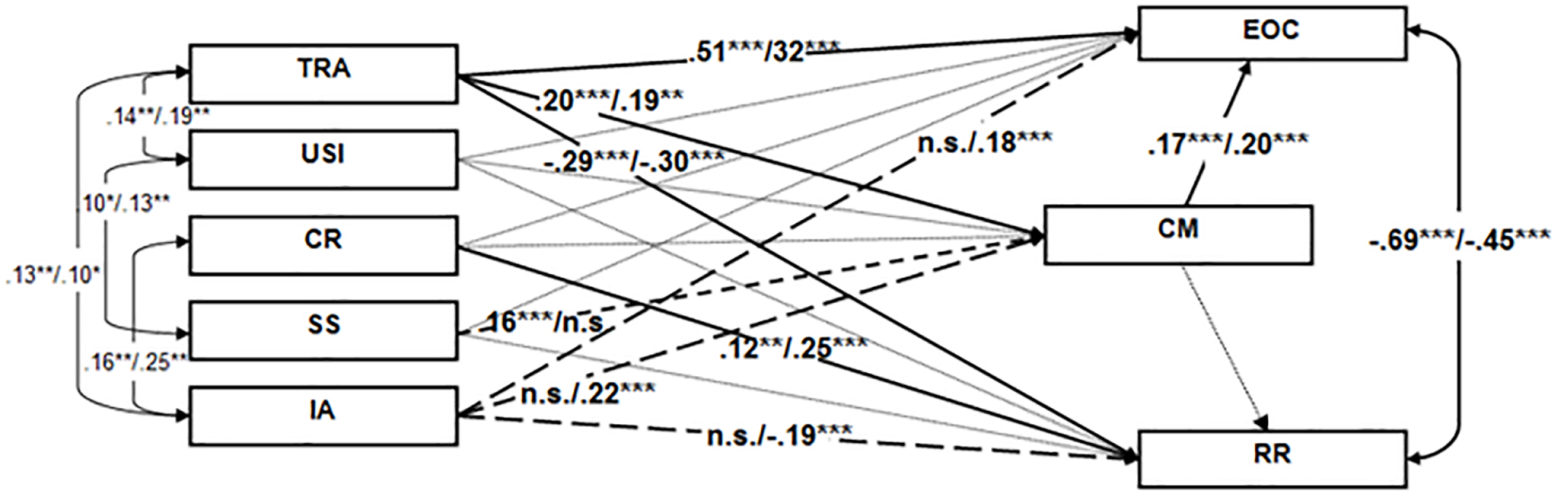
Conspiracy mentality as a partial mediator of the relations between Social Attitudes and Ethos of Conflict and Readiness for Reconciliation. *Note.* RR = Readiness for Reconciliation; EOC = Ethos of Conflict; TRA = Traditional Religiosity; USI = Unmitigated Self-Interest; CR = Communal Rationalism; SS = Subjective Spirituality; IA = Inequality-Aversion; CM = Conspiracy Mentality. Significant paths are presented with: solid lines if they are significant on both subsamples, dash lines if they are significant only on the Serbian sample, and long dash lines if they are significant only on the Kosovo sample. Nonsignificant paths are depicted with square dot lines. Standardized regression coefficients are presented and separated by a slash between Serbia/Kosovo. *p < .05. **p < .01. ***p < .001.

We have tested the hypothesis that Conspiracy Mentality mediates the relationships between basic social attitudes (set as exogenous variables in the model) and both Ethos of Conflict and Readiness for Reconciliation (set as endogenous variables) (Figure 1).

To test cross-sample stability, we have employed multi-group analyses. Three models with increasing strictness were tested: the configural invariance model, the path invariance model, and the error variances invariance model. The configural invariance model assumes the invariance of the structure of relations between the variables (Horn & McArdle, 1992). The path invariance model assumes that the paths from the predictors to the criterion variables are the same across the two samples. Finally, the error variances invariance model assumes the error variances of the variables to be equal across the two samples. The method of parameter estimation was a maximum likelihood. The results, displayed in Table 4, showed that none of these three models are invariant.

**Table 4:** Model Testing Cross-Sample Stability

Model	χ^2^	*df*	RMSEA	90%CI	CFI	Δχ^2^	*df*
Configural (baseline) invariance	29.50	26	.02	.00–.04	.99		
Path invariance	50.17	36	.03	.00–.04	.98	20.67*	10
Error variances invariance	441.64	57	.08	.07–.09	.92	412.14**	31

*p < .05. **p < .01.

The model presented in Figure 1 showed that the expected direct pathways of Traditional Religiosity and Communal Rationalism toward Ethos of Conflict and Readiness for Reconciliation respectively can be detected. Traditional Religiosity had a direct positive loading on general Ethos of Conflict, and a direct negative one on Readiness for Reconciliation. Communal Rationalism positively influences the Readiness for Reconciliation. Except for these two, only the direct paths of Inequality-Aversion towards both of the reconciliation measures are registered. Conspiracy Mentality positively influences Ethos of Conflict, but not Readiness for Reconciliation. The model shows that Traditional Religiosity has an indirect effect, mediated by Conspiracy Mentality, on the Ethos of Conflict. Conspiracy Mentality mediated the relationships between Subjective Spirituality and general Ethos of Conflict on the sample from Central Serbia, and Inequality-Aversion on the sample from Northern Kosovo.

## Discussion

This study has aimed to investigate the relations between Conspiracy Mentality, basic social attitudes, and psychological factors that could either hinder or facilitate reconciliation between groups in conflict. The results showed that there were significant differences between subsamples of Serbs from Central Serbia and Northern Kosovo on almost all examined variables, including Conspiracy Mentality. Respondents from Kosovo showed more conservative, pro-conflict, selfish and materialistic attitudinal orientations. Respondents from Central Serbia were more pronounced toward conspiracionism, but also more oriented toward humanistic values and intergroup reconciliation. Besides that, Conspiracy Mentality and Traditional Religiosity were negatively related to the Readiness for Reconciliation and positively to the Ethos of Conflict. In addition, Conspiracy Mentality made an independent contribution to understanding the Ethos of Conflict over and above basic social attitudes. Finally, Conspiracy Mentality mediated the relations of Traditional Religiosity, Subjective Spirituality and Inequality-Aversion with the Ethos of Conflict (but not Readiness for Reconciliation). The results are relatively consistent throughout the samples and mainly supportive for the hypotheses considered.

### Conspiracy Mentality and its Relation to Basic Social Attitudes and the Psychological Foundations for Reconciliation

This study provides new evidence that conflict beliefs should emerge from the conservative attitudinal orientation. As expected, the results showed that the Serbs from Northern Kosovo are more conservative than those from Central Serbia (higher Traditional Religiosity and Unmitigated Self-Interest, lower Communal Rationalism). They also exhibit more pronounced indicators of Ethos of Conflict or Readiness for Reconciliation with the Albanians than do Serbs from Central Serbia with the Croats. These results are in line with previous findings of differences in attitudinal orientation and Ethos of Conflict between Serbs from Central Serbia and Northern Kosovo (Međedović & Petrović, 2012). However, it is interesting that Serbs from Northern Kosovo have a less pronounced Conspiracy Mentality than Serbs from Central Serbia, although respondents from both subsamples scored relatively highly on Conspiracy Mentality. This finding is also in line with the studies which have argued that Conspiracy Mentality could have an important role in a conflict situation, strengthening and justifying hostility toward out-groups (Pruitt, 1987; Sedek & Bilewicz, 2015). Conspiracy Mentality facilitates the perception of an out-group threat, even if there are no objective reasons for it - this is the reason why Conspiracy Mentality is more pronounced within the subsample that is focused on the (still latent) conflict with the Croats than within the subsample more focused on the (still ongoing) conflict with the Kosovo Albanians (Bergmann, 2018). More interestingly, the results support the hypothesis that war exposure may have even a long-term impact on radicalisation (see, for example, Vlachos, 2016).

A somewhat more complete picture can be obtained by looking at the relationships between the examined variables. As expected, Traditional Religiosity is positively related to the Ethos of Conflict and negatively to the Readiness for Reconciliation, but Communal Rationalism is positively associated with the Readiness for Reconciliation. These findings are in line with previous studies, showing that political conservatism is positively related to out-group hostility and preferences inclining towards aggressive actions against the out-group (De Zavala, Cislak, & Wesolowska, 2010), and to Ethos of Conflict (Petrović & Međedović, 2011; Međedović & Petrović, 2013, 2016), and negatively to Readiness for Reconciliation (Petrović, 2005). It is important to emphasize that these findings are stable throughout the subsamples, and supported by regression analysis.

Besides this, we found the findings related to Inequality-Aversion and its associations with the other variables very interesting. Inequality-Aversion (i.e., Egalitarianism) is positively associated with Communal Rationalism, in line with theoretical expectations, but is also positively related to Traditional Religiosity and Ethos of Conflict, and negatively to Readiness for Reconciliation. These findings are theoretically unexpected, but congruent with some previous findings (e.g., Petrović, 2016). Although surprising, these findings suggest the same kind of mixture of egalitarianistic and conservative ideology which has been recognized in Serbia from the beginning of the 1990s and labeled as socialistic conservatism (see, for example, Todosijević, 2006). It is difficult here to resist failing to mention the concept of left-wing authoritarianism, which, evidently, is not the myth of the post-communist countries (de Regt, Mortelmans, & Smits, 2011). It is interesting to note, though, that Inequality-Aversion is positively related to Conspiracy Mentality, and significantly contributes to the prediction of Readiness for Reconciliation (negatively) and Ethos of Conflict (positively), but only in the Kosovo sample. These findings can be understood if we have in mind that some studies have shown that both right- and left- political extremists are inclined to believe in conspiracy theories (Krouwel et al., 2017).

Similarly to social attitudes, Conspiracy Mentality has a moderate positive relation to the Ethos of Conflict and a weak negative relation with the Readiness for Reconciliation. More importantly, Conspiracy Mentality independently positively contributed to the prediction of Ethos of Conflict over and above Traditional Religiosity, as well as negatively to the prediction of the Readiness for Reconciliation over and above Traditional Religiosity and Communal Rationalism, but only in the Serbian sample. These findings are in line with our second hypothesis and with previous studies which have also shown positive relations between conservative social attitudes, primarily authoritarianism, and the propensity to belief in conspiracy theories (Bruder et al., 2013; Richey, 2017). However, we should bear in mind that Conspiracy Mentality and Ethos of Conflict are conceptually similar and share some important characteristics: they originate during societal crises (van Prooijen & Douglas, 2017), reflect the need to control the environment (Douglas et al., 2017), foster a positive self-view about the in-group (Cichocka et al., 2016), derogate out-group members (Mashuri & Zaduqisti, 2014), etc. We should acknowledge some attempts to consider Conspiracy Mentality as an important psychological factor contributing to the escalation and maintenance of intergroup conflicts (see, for example, Pruitt, 1987). The results of our study are in line with these theoretical expectations, but additional empirical testing is needed.

### Conspiracy Mentality Mediates the Link Between Basic Social Attitudes and Ethos of Conflict

There is a tendency in the literature referring to conspiracy theories that significant efforts are being made to understand the nature of the proneness to believe in conspiracy theories and its antecedents (see, for example, Bruder et al., 2013; Mashuri & Zaduqisti, 2014; Richey, 2017). But it seems that less attention has been paid to the investigation of the role of Conspiracy Mentality in understanding other socio-psychological constructs. Bearing in mind that post-Yugoslav societies are still in the post-conflict period, the issue of reconciliation is of crucial importance. These are the reasons why this research has aimed to investigate the potential role of the Conspiracy Mentality in explaining the Ethos of Conflict and Readiness for Reconciliation as a key psychological factor in the processes of promoting (or hindering) reconciliation.

The hypothesis that Conspiracy Mentality will mediate the relations between social attitudes and both Ethos of Conflict and Readiness for Reconciliation, was partly confirmed. Results of multi-group path analyses showed that, stable throughout the samples, Traditional Religiosity and Communal Rationalism have direct effects on conflict beliefs, and, consequently, on the Readiness for Reconciliation: Traditional Religiosity is a predisposition for the emergence of conflict beliefs and for prevention of reconciliation, whilst Communal Rationalism, which reflects trust in democratic institutions, could facilitate positive views on reconciliation. What is to be noted here is that Conspiracy Mentality mediates the effects of Traditional Religiosity on Ethos of Conflict and Readiness for Reconciliation, and these effects are also registered in both samples. These findings provide support for the view that conservative people are generally prone to conflict beliefs (Međedović & Petrović, 2016). But the multigroup analysis showed that two samples differed regarding the effects of Subjective Spirituality and Inequality-Aversion on the Conspiracy Mentality and both reconciliation-relevant variables. Namely, only in the sample from Central Serbia is the effect of Subjective Spirituality on Ethos of Conflict moderated by the Conspiracy Mentality. This finding supports the view that conservative attitudes and proneness to unusual thinking and spirituality facilitate beliefs in conspiracy theories (see, for example, Darwin, Neave, & Holmes, 2011), which further contribute to the development and maintenance of conflict-oriented beliefs (see, for example, Pruitt, 1987). When the effects of Inequality-Aversion are considered, the results show that Inequality-Aversion has both direct and indirect effects, i.e., Inequality-Aversion hinders Readiness for Reconciliation, but facilitates conflict-oriented beliefs, supported by Conspiracy Mentality. This pattern of relations is practically identical with the pattern which Traditional Religiosity provides. We discussed these findings earlier, which were theoretically unexpected but consistent with some previous research, with reference to concepts of socialistic conservatism (Todosijević, 2006) or left-wing authoritarianism (de Regt et al., 2011). Still, it is interesting that these effects are registered only in the sample of Serbs from Northern Kosovo but not of those from Central Serbia. Following the previous literature on the subject (Međedović & Petrović, 2012), it could be assumed that closeness to the conflict zone leads to the radicalisation of both right-wing and left-wing authoritarians (Krouwel et al., 2017), i.e., right-conservative and socialistic-conservative people (Todosijević, 2006). This becomes even clearer if we bear in mind that both left-wing and nationalistic right-wing political parties in Serbia (e.g., the Socialist Party of Serbia and the Serbian Radical Party, respectively) have been saturated with the same principal stable component throughout the period (Petrović & Međedović, 2017). More precisely, from the beginning of the 1990’s, Slobodan Milosević's Socialist Party of Serbia and Vojislav Šešelj’s Serbian Radical Party were the dominant political forces in Serbia. Consequently, these two parties were the bearers of the responsibility for the overall social changes during the 1990’s, including the escalation of the conflict between Serbs and other Yugoslav nationalities. It is important to note here that these parties, although formally ideological opposites, essentially are not: both of them have been supported mainly by working-class voters, and both of them are characterised by a mixture of left-wing (i.e., social justice) and extreme nationalistic rhetoric (Bakić, 2015). After the fall of Milošević in 2000 and the democratic reforms in Serbia, it is interesting that both of these parties are dominant political forces in Northern Kosovo, in contrast with Central Serbia^ii^.

In this context, it becomes understandable why politicians and policy-makers in conflict and post-conflict periods have established strong conflict narratives (Petrović, 2017), and also promoted and propagated different conspiracy theories. For example, during the 1990s, the Serbian regime supported the development and maintenance of different conspiracy theories seeking to justify the dissolution of Yugoslavia and the role of Serbia in the ensuing conflicts (Byford, 2002; Byford & Billig, 2001). It is additionally important to notice that these conspiracy theories supported the dominant narrative among the Serbian people - the narrative about the Serbs (and Serbia) as historical victims, with the continuous need to defend themselves from external threats, including from the international community, which is seen as negative, unjust and unfair towards the Serbian people (Byford, 2006; Obradović & Howarth, 2018). This narrative is typically recognized as the „national siege mentality“, the core societal belief of members of one society that the rest of the world have negative intentions towards them (Bar-Tal & Antebi, 1992). Previous research has shown that about one-third of citizens in Serbia, primarily voters for the Serbian Radical Party, express a high degree of national siege mentality (Šram, 2009), which is one of the preconditions for the development of conflict-oriented beliefs (Bar-Tal, 2007). Therefore, the findings of this research indicate that it is important to reduce the propensity to believe in conspiracy theories if conflict-oriented beliefs are to be decreased.

### Limitations and Future Directions

In conclusion, we should point to some of the limitations and unresolved issues of this study. Firstly, the current research is based on cross-sectional data; this has prevented us from inferring causal relations from the data and led us potentially to under- or over-estimate the longitudinal effects. Longitudinal research can only provide the estimations of the causal links between examined variables. Second, the results showed that there are several Cronbach’s alpha coefficients for some of the measurements (e.g., Subjective Spirituality and Inequality-Aversion), suggesting that the measurements were somewhat unreliable. Also, it must be noted that reliabilities are generally slightly weaker in the Kosovo subsample than in the subsample from Central Serbia. Although some coefficients of reliability are lower, they are still acceptable, but it can be assumed that this could have affected the results of the study and that therefore the results must be interpreted with a certain caution (Sawilowsky, 2009). Third, this research was focused only on Serbian respondents, and it would be important to test the replicability of the findings on the other ethnicities with whom the Serbs were in conflict. Fourth, we should note that respondents from Central Serbia were not living in the war zone in the 1990s, as was the case with respondents from Kosovo at that time. Therefore, future studies should include participants of Serbian nationality from the conflict zone in Croatia. Fifth, the samples consisted of both students and their parents, which could have affected the results of the study, having in mind its nestedness. However, we believe that this constraint does not affect the results of this study, because some previous research has shown that there were no differences between students and parents with regard to social attitudes and Ethos of Conflict (Međedović & Petrović, 2018). The finding that family structure had no effect in regression models in this study also confirmed our assumption regarding the structure of the samples. Sixth, this study resulted in one unexpected result: in the Kosovo sample, Inequality-Aversion (i.e., Egalitarianism) and Traditional Religiosity have identical patterns of relations with the Ethos of Conflict, Readiness for Reconciliation and Conspiracy Mentality (which mediates their relations). We have postulated several explanations for these findings: hypotheses about left-wing authoritarianism, about the dominance of political parties with both nationalistic and socialistic ideologies, and about the role of environmental harshness (closeness to the conflict zone) facilitating both right- and left- wing extremism and their relations with Conspiracy Mentality. These findings and their possible interpretations are certainly intriguing, and further research might confirm them and provide a clearer framework for their interpretation. Seventh, the results of our study, consistently with those previous to ours (e.g., Bar-Tal et al., 2012; Petrović & Međedović, 2011), showed that eight conflict beliefs constitute the unidimensional General Ethos of Conflict in each of the subsamples. However, it is important to note that these eight beliefs have different loadings to the General Ethos of Conflict in the different subsamples, which suggested a potentially different structure of the Ethos of Conflict and its dependence on contextual factors, i.e., on the specifics of inter-group conflict. It is important to note that previous studies have also shown that the structure of the Ethos of Conflict, but also the content of conflict-oriented beliefs could be changed over time and as a consequence of the major events that characterise conflicts (Oren, 2009). We do not expect that the General Ethos of Conflict, regardless of the potential differences in its structure in various (post)conflict societies, will have different relations with external measures in dependence of its structure. But the insight into the structure of the Ethos of Conflict in different societies could be important for the deeper understanding of the Ethos of Conflict itself, and also of the nature of a specific inter-group conflict. Finally, we should mention that the instrument used for assessment of Conspiracy Mentality, i.e., CMQ, is a general measure of the propensity to believe in conspiracy theories, which to a certain extent reflects some kind of political cynicism (Milošević-Đorđević & Žeželj, 2017b). It might be that the effects would be even more powerful if some more adequate, more specific measure of conspiracy theories was used.

### Conclusions

In line with our hypotheses, the results of this study suggest that, in post-Yugoslav countries, the general propensity to endorse conspiracy theories facilitates the possibility of developing conflict-oriented beliefs, especially with conservative people. It seems that Conspiracy Mentality, i.e., the disposition toward belief in conspiracy theories, could be an important socio-psychological factor hindering reconciliation and facilitating prolongation of intergroup conflicts. This might be the reason why Conspiracy Mentality, together with other important constructs like basic social attitudes, should be taken into consideration when creating policies and programmes focused on reconciliation between groups after violent conflicts.

Efforts to determine the socio-psychological factors that could promote reconciliation in (post)conflict societies might result in the development of specific interventions to reduce conflict beliefs and to promote peace (see, for example, Halperin & Sharvit, 2015; Hameiri, Nabet, Bar-Tal, & Halperin, 2018). The studies mentioned showed that these interventions could have significantly reduced conflict beliefs even in hawkish (conservative, right-wing) people who are closed-minded and rigid in their hostile attitudes toward out-groups (Hameiri, Porat, Bar-Tal, & Halperin, 2016). Similarly, Swami and colleagues (Swami, Voracek, Stieger, Tran, & Furnham, 2014) showed that stronger beliefs in conspiracy theories were associated with lower analytic thinking, lower open-mindedness and greater intuitive thinking, and proposed an analytic-thinking based intervention programme for reduction of conspiracy beliefs. The results of our study also have practical implications, since it was demonstrated that Conspiracy Mentality has an important role in hindering reconciliation. Our results indicate that in the case of people with more conservative attitudes, an intervention programme based on the elicitation of analytic thinking could be a useful tool to decrease conspiracy and conflict beliefs and to promote reconciliation between former-Yugoslavian nations.

## Appendix

**Table A1:** Results of Principal Component Analysis of the Readiness for Reconciliation Subscales on the Whole Sample and Serbian and Kosovo Subsamples

Characteristics of the PCA	Serbia		Kosovo		Whole sample	
	λ_1_	λ_2_	λ_1_	λ_2_	λ_1_	λ_2_
Random data	1.12	1.03	1.14	1.04	1.09	1.02
Real data	2.56	.63	2.11	.78	2.50	.61
% of the explained variance	64.1		52.7		62.6	
Trust	.65		.49		.64	
Cooperation	.86		.72		.82	
Forgiveness	.74		.78		.78	
Rehumanization	.64		.45		.58	

**Table A2:** Results of Principal Component Analysis of the Ethos of Conflict Subscales on the Whole Sample and Serbian and Kosovo Subsamples

Characteristics of the PCA	Serbia		Kosovo		Whole sample	
	λ_1_	λ_2_	λ_1_	λ_2_	λ_1_	λ_2_
Random data	1.24	1.15	1.26	1.16	1.17	1.10
Real data	3.51	1.14	2.65	1.13	3.65	.91
% of the explained variance	43.9		33.1		45.6	
Justification of goals	.18		.69		.59	
Beliefs about security	.61		.48		.65	
Delegitimization of the opponents	.79		.75		.80	
Patriotism	.50		.30		.50	
Beliefs about unity	.44		.30		.47	
Positive collective self-view	.72		.34		.64	
Victimization	.76		.67		.71	
Beliefs about peace	.52		.20		.51	
